# Extending the science fair project beyond the walls of the gymnasium with the Canadian Science Fair Journal

**DOI:** 10.1038/s42003-019-0623-z

**Published:** 2019-10-09

**Authors:** Rhiannon Ng, Kira Slivitzky, Richard Webster, Dayre McNally

**Affiliations:** 1 Children’s Hospital of Eastern Ontario Research Institute, 401 Smyth Road, Ottawa, ON Canada; 2 Division of Critical Care, Department of Pediatrics, Faculty of Medicine, University of Ottawa, Ottawa, ON Canada

**Keywords:** Authorship, Education

## Abstract

It is increasingly recognized that research is most impactful when disseminated to broad audiences within and beyond the scientific community. For children and youth, opportunities to share independent research beyond family and science fair attendees are limited by a lack of appropriate dissemination platforms. This lack of opportunity creates the ‘science fair dilemma’, where the engagement of students with the scientific community is curtailed once science fairs wrap up. Here we discuss this missed opportunity to encourage engagement and skill development of young scientists, and provide a case study of a student centric science journal aimed to tackle these challenges.

## The science fair dilemma

The science fair is a long-held tradition across educational curricula, and has been heralded as a learning tool that promotes longitudinal interest and self-efficacy in Science, Technology, Engineering, and Mathematics (STEM)-driven youth^1,2^. Dating back to 1942 when an American program established the high school science fair as a talent search strategy, science fairs have since expanded into prestigious events that reach the level of global competition level^3^. Every year, elementary and high school students from around the world learn to navigate the waters of scientific research by undertaking independent projects on topics of their choosing. In Canada alone, 25,000 children and youth from across the country participate in regional science fairs on an annual basis, with ~500 participating in the national fair^4^. Drawing upon weeks to months of rigorous experimentation, data collection, and prototype refinement, students attend science fairs with the hope of sharing their interests and connecting with like-minded individuals. From biomedical computing and diabetes treatments to reusable energy and autonomous vehicles, the scientific inquiries conducted by today’s young scientists span a vast range of topics and often address pressing scientific and societal issues^5^. Needless to say, science fairs have evolved into critical platforms for young scientists to showcase their research and exercise their role in empowering other STEM-driven students.

However, the youth science culture is evolving rapidly with a growing number of students participating in science fairs, increasingly diversified and relevant research topics, and a shift toward digitized knowledge. In light of the shifting landscape of youth science, there are aspects of the science fair model that can and should be improved to enhance the experience. Consider Sasha, a 12-year-old student from rural Nunavut; after months of trialing methods, collecting data, and testing results at her home kitchen sink, she developed a sustainable method for converting potato peels into usable bioplastics. She devised a poster board illustrating her project from inception to conclusion, and went on to compete first regionally, then nationally at the Canada-Wide Science Fair (CWSF). At the national competition, Sasha met several other students who, like herself, had spun their original ideas and experiences into highly impactful scientific research projects. Then, competition completed, Sasha folded up her poster board and returned home to Nunavut. With no further use for it, she left it on the kitchen counter, until someone moved it to her bedroom, until she moved it to the basement, where it stayed until…Well, where it stayed. When projects such as Sasha’s go into storage to collect dust, they miss the opportunity to further benefit their creators, other students, and perhaps the world.

The fundamental issue with the science fair model is its lack of continuity. While the fair itself provides the physical space for young scientists to showcase their work, platforms for publishing science projects are limited. Indeed, in a study conducted by Canadian researchers, <8% of CWSF medalists go on to publish their work (Acharya et al. (manuscript in preparation)). This 8% publication rate is likely a substantial over-estimation for all children and youth, considering this sample focused on national winners, and excluded those at school fairs and regional competitions. Without a developmentally appropriate medium for students to disseminate their research after the fair, the majority of science fair projects have longevities confined to either a single day or a few short weeks.

## The value of scientific publication for youth

Scientific publication has evolved into a tradition across disciplines and research communities, and is now recognized as a critical component of the research process. It is widely accepted that science involves publishing and communicating findings with others, with many research funders requiring publications and knowledge translation as key deliverables. When it comes to research conducted by children and youth, these contributions range from anecdotal analyses of the effects of social media on anxiety to the development of forest fire detectors – they are original, innovative, and address real-world problems. In failing to provide students with a platform for disseminating their work beyond science fairs, we allow for these valuable contributions to slip through the cracks. Not only do students miss the opportunity to share their work, but they are also denied the far-reaching benefits of publication. The process of publishing opens doors for scientists to engage with like-minded researchers, network with professionals, and build credit. In addition, and particularly for younger students, scientific publication allows for authors to enhance their writing skills, gain experience with peer review, and develop a critical eye for research appraisal. By excluding youth from the benefits of widespread dissemination, we do a disservice not only to these future scientists, but to the scientific community.

## From posterboard to publication: *Canadian Science Fair Journal* case study

In our investigation of publication rates in CWSF winners, we found that one of the primary reasons cited by Canada’s youngest researchers preventing publication of their work was the absence of an appropriate platform^4^. To combat the lack of accessible media for the dissemination of youth science, our team founded a journal dedicated to showcasing science projects by Canadian children and youth. Launched in 2018, the *Canadian Science Fair Journal* (*CSFJ*) is an open-source, peer-reviewed publication that features the work of students aged 6–18 from across Canada (www.csfjournal.com). The *CSFJ* mandate is to promote science literacy in younger populations by providing indiscriminate access to publication opportunities for Canadian youth. Upon announcing its launch, the influx of submissions and excitement generated in science fair winners, educators, and professional scientists across the country only affirmed the need for a journal dedicated to showcasing youth research.

**Fig. 1:**
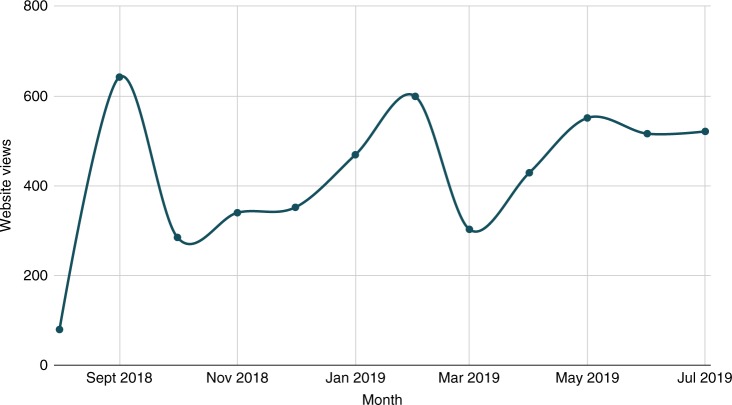
Website views per month over the *CSFJ*’s inaugural year. Online issues were published in September, February, and May, at which times website traffic increased

Over the course of its inaugural year, *CSFJ* published 3 issues featuring the work of students hailing from the tip of the Northwest Territories to coastal Prince Edward Island (2800 miles apart) and from many small towns and urban centers in between. The manuscripts written by these students are original and innovative; one student developed a sustainable carbon dioxide capture system using household materials and algae, another performed a statistical analysis of suicide in Canada, and yet another built a transportation device to reduce mortality rates in baby chicken shipments. Each article was published both online and in print, with print issues being disseminated to authors, schools and libraries across the country. The necessity in providing students with this experience was reflected in their feedback, with several authors expressing excitement at the opportunity to learn about the mechanics of publication while building research credit and sharing their work with larger audiences. One author claimed that “The *CSFJ* is now an essential part of the Canada-Wide Science Fair and school experience in Canada”, and was one of several students who re-submitted their most recent projects for the journal’s second publication year. Student editors have also commented on the value this experience brings both to their mentees and to themselves. In a letter to the Editor-in-Chief, one of the journal’s original editors stated that having the opportunity to mentor young scientists through the publication process had “reinforced [their] own scientific knowledge and commitment to engaging in research and scientific education”. Further, educators across Canada have been requesting issues and using the journal in their classrooms to teach scientific principles and inspire their own students. As articles were released and readership expanded, so too did the journal’s online community; in <1 year, the initiative garnered over 600 collective followers on its social media pages, with students, educators, and scientists from Canada and beyond showing virtual support for the initiative. Over its first year, the journal’s website also garnered significant traffic, reaching a cumulative 14,000 views in less than a year after its launch. While overall traffic to the website (i.e. paper reads) has been a success, we have observed significant fluctuations over time, with prominent peaks corresponding to the three online publication dates followed by lulls in between (Fig. 1). The positive uptake shown over the *CSFJ*’s inaugural year validated the need for a platform that brings the young STEM community together while recognizing the work of our next generation of scientists.

As is the case with new projects, the journal encountered barriers that prompted us to re-evaluate and reshape its framework over the year. As an example, for volume 1, issue 1 the journal initially directed authors to submit their work through a third party journal management software (Scholastica). However, while acceptable to more senior scientists, the step proved daunting to students new to the realm of scientific publication, and acted as a barrier to participation. Submission volume increased rapidly after we modified the submission process to allow authors to submit their work through simple email. This shift spoke to the importance of minimizing superfluous learning curves within a process that is already full of new experiences for first-time authors. In addition, we found that tri-annual publication resulted in sporadic readership volume, as depicted in the figure illustrating website views over the year. With the goal of achieving a more consistent level of engagement and promoting continuity in public appreciation for youth science, the journal has made the decision to release articles online on a monthly basis. As one final example of lessons learned, the journal did not receive French submissions during the inaugural year, which was a point of concern given the journal’s mandate to promote an inclusive science in a bilingual country. Moving into year two, the journal took steps to expand language accessibility for Canadian students by translating its website and training French-speaking editors. In 2019 several French language submissions were received and will be published in its upcoming volume.

**Fig. 2:**
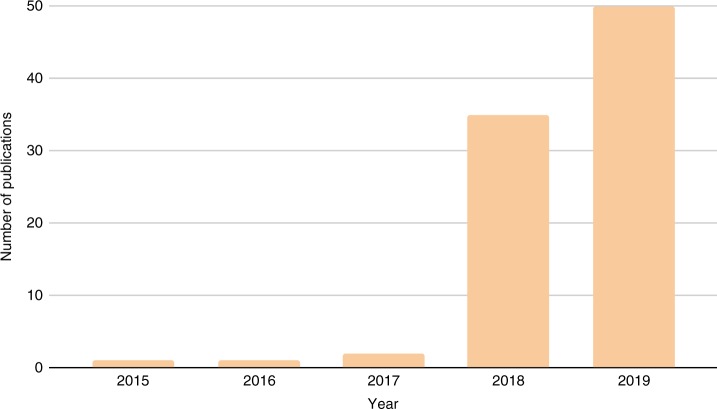
Number of Canada-Wide Science Fair projects published per year. The *CSFJ* launched for its first call for submissions in 2018

Now entering into its second year, the journal has continued to grow in the number of submissions from science fair competitors from both inside and outside of Canada (Fig. 2). This growing interest in publication may indicate that students from other countries are seeking similar opportunities, and further supports the need for publication platforms for STEM-driven children and youth across the global research community. Intriguingly, 20% of the journal’s website traffic originates from outside Canada, with a strong readership in Asia and Europe. This rapidly increasing interest in youth science publications may be yet another call to action to expand the number and breadth of similar platforms around the world.

## Opening doors for young scientists

The benefits to scientific publication are widely acknowledged amongst the research community. Publication allows students not only to disseminate their work broadly, but also to experience the benefits of having their work shared in a formal, open-source platform. Arguably one of the most valuable benefits of early publication is exposure to concepts of scientific writing. The *CSFJ*, for instance, aspires to accomplish this through an editorial board comprised of university students specializing in a variety of fields. Unlike the traditional peer review process, these editors mentor authors through the editorial process while providing them with constructive feedback and introducing them to important concepts in science writing. In providing students with valuable experience in editing and critical appraisal, publication platforms can take steps to demystify the publication process and better equip these young scientists for their future careers.

In addition to writing experience, youth science journals can act as catalysts in helping authors make professional connections through their published work. One author recently published in *CSFJ* was contacted by Dr. Cherisse Du Preez, a marine biologist, who had read her article. Dr. Du Preez featured this author in a presentation and went on to give her a tour of the Ocean’s Institute in Vancouver, Canada. Other authors have had their articles shared on prominent scientists’ social media accounts and personal blogs, and continue to make new connections within the broader research network. As youth publication rates increase, it is becoming ever more important that we recognize youth publication as an important aspect of career development.

## Giving children and youth a louder voice in the research world

While scientific publications enhances students’ professional credit and grants them a foothold in the research world, its benefits extend far beyond the agenda of career enhancement. In walking down rows of poster boards at a national science fair competition, it is hard to miss the common thread of science advocacy amongst the work of today’s young scientists. With topics ranging from environmental damage and renewable energy to learning disabilities and adolescent anxiety, many youth scientists are unafraid to push important topics to the forefront of conversation and tackle them using their passion for science. There is an underlying urgency to the discourses young scientists bring to the table, and their work is proof of their pro-activity and willingness to take action. For instance, 41% of articles published in *CSFJ*’s inaugural issue addressed climate change, and several others investigated issues such as bullying, access to potable water, and adolescent anxiety. More importantly, the youth research surrounding these issues often stems from a place of lived experience and observation. In Canada, Indigenous students competing at science fairs often conduct projects focused on environmental damage and their traditional relations with the land – a pressing concern within Indigenous communities in Canada. It is clear that these scientists are assuming accountability – even if it is not their obligation – and taking strides to reshape the world in which they live. Youth publication can thus play a critical role in promoting epistemic diversity and lending representation to scientists stemming from diverse social vantage points – whether those be cultural, socioeconomic, or geographical. In providing these students with a platform to disseminate their work, amplify their voices, and make their presence known, publication platforms do justice to their investments and experiences while promoting an inclusive, representative science.

## Where do we go from here?

Youth science is expanding in its complexity, impact, and relevance. It is the responsibility of scientific and education communities to provide children and youth with accessible platforms to disseminate their research. Not only do students benefit professionally from scientific publications, but it also gives these young academics a platform for their voices to engage in advocacy. The interest generated by *CSFJ* in its first year alone speaks to the need for more publication platforms targeting youth science beyond Canada. However, the role of science journals extends far beyond research dissemination. The *CSFJ* is currently developing tools for educators to use in the classroom to teach concepts of science literacy and promote STEM education in the general population. Publication platforms targeting youth science can serve as resources for educators to use the scientific research performed by youth to teach peers from similar or divergent backgrounds, interests, and geographies. Consequentially, the impacts of publication can transcend the research community itself and serve as valuable tools for enhancing education in all children and youth. There is undoubtedly a need for more youth science representation in the publishing world. It is time that academic publishing houses, established researchers, editors, and educators around the world take steps to promote youth science in the form of publications to support the success of our next generation of scientists.
